# Supplementary material for: Association between body mass index and blood pressure levels across socio-demographic groups and geographical settings: analysis of pooled data in Peru
Source: PeerJ. 2021 Apr 21;9:e11307. doi: 10.7717/peerj.11307 (PMC8067913; doi:10.7717/peerj.11307)
Supplement: Supplemental Information 1 [file peerj-09-11307-s001.docx]

# Supporting information

## Statistical approach: Modeling the association between body mass index and blood pressure levels.

The relationship between body mass index and systolic (SBP)/diastolic (DBP) blood pressure levels has been largely described in the literature; and therefore, different models have been created to account for this association.

In this manuscript, we have fitted a non-linear regression model with specific coefficients for our outcomes and exposure of interest (i.e., SBP/DBP and BMI). Thus, initially we fitted a crude regression model including a quadratic term for BMI as follows:

Y = β_0_ + β_1_.BMI + β_2_.BMI^2^ (1)

Where Y was our outcome (systolic or diastolic blood pressure depending on the model), β_0_ was the intercept (i.e., the mean blood pressure when BMI is equal to 0), β_1_ was a coefficient indicating the average change of blood pressure by one-unit change in BMI, and β_2_ was the change in blood pressure levels due to a change in a squared-unit change in BMI. However, for our estimations, we used an adjusted model that includes age as continuous variable and its quadratic form as well as other sociodemographic variables as categorical variables. Thus, our adjusted model was:

Y = β_0_ + β_1_.BMI + β_2_.BMI^2^ + β_3_.AGE + β_4_.AGE^2^ + β_5_.SOCIODEM (2)

In model 2, we have introduced two coefficients for assessing age: β_3_ as age and β_4_ as the quadratic term of age. In addition, we included also β_5_ as a coefficient for a matrix of sociodemographic categorical variables (i.e. a specific coefficient for sex, education level, socioeconomic position, study area, and altitude).

Finally, when the effect modification of any of the sociodemographic variables was evaluated, we fitted a slightly different model using a similar structure as follows:

Y = β_0_ + β_1_.BMI*SEX + β_2_.BMI^2^*SEX + β_3_.AGE + β_4_.AGE^2^ + β_5_.SOCIODEM (3)

Using the variable sex as an example (model 3) and based on the hierarchical approach, the sociodemographic variable was introduced in the adjusted model as an interaction term multiplying the BMI and the quadratic term of BMI. Then, the sex variable was excluded from the matrix of sociodemographic characteristics of the β_5_ coefficient. However, in the specific case of age, our model 3 did not exclude age neither the quadratic term of age as continuous variable from the model. This was done to allow for a better modeling into each age category as these categories were relatively wide.

Overall, effect modification was assessed by the Wald test using the *testparm,* a post-estimation command in STATA. P-values <0.05 were considered significant.

The Theorem of Central Limit states that in a population with mean μ and standard deviation σ with sufficiently large random samples from the population, the distribution of the sample means will be approximately normally distributed. This assumption was used to build our models. However, we created a non-linear regression model because we expected the association was not lineal.

Collinearity was evaluated using the tolerance test in STATA as described previously (https://stats.idre.ucla.edu/stata/faq/how-can-i-check-for-collinearity-in-survey-regression/).

**Table S1.** Overall association between body mass index and blood pressure:

adjusted models

| **Model variables** | **Systolic blood pressure** | **Diastolic blood pressure** |
| --- | --- | --- |
|  | **β (95% CI)** | **β (95% CI)** |
| BMI | 2.36 (2.14; 2.58) | 1.47 (1.33; 1.61) |
| BMI^2^ | -0.03 (-0.03; -0.02) | -0.02 (-0.02; -0.01) |
| Sex (male vs. female) | 9.89 (9.61; 10.16) | 4.85 (4.69; 5.02) |
| Age | -0.29 (-0.35; -0.23) | 0.45 (0.42; 0.48) |
| Age^2^ | 0.01 (0.01; 0.01) | -0.01 (-0.01; -0.01) |
| Education |  |  |
| 7-11 vs. <7 years | -0.25 (-0.68; 0.17) | 0.56 (0.34; 0.78) |
| 12+ vs. <7 years | -1.15 (-1.63; -0.67) | 0.82 (0.55; 1.08) |
| Socioeconomic position |  |  |
| Middle vs. low | -0.61 (-0.99; -0.22) | -0.59 (-0.83; -0.36) |
| High vs. low | -0.06 (-0.55; 0.44) | -0.20 (-0.50; 0.10) |
| Area (urban vs. rural) | 0.96 (0.57; 0.44) | 0.79 (0.54; 1.04) |
| Altitude |  |  |
| 501-2500 vs. <500 m.a.s.l. | -0.86 (-1.23; -0.48) | 0.63 (0.40; 0.87) |
| 2501+ vs. <500 m.a.s.l. | -1.87 (-2.18; -1.55) | 1.67 (1.46; 1.87) |
| Year | -0.08 (-0.17; 0.00) | 0.18 (0.13; 0.23) |

**Table S2.** Association between body mass index and blood pressure by sex:

adjusted models

| **Model variables** | **Systolic blood pressure** | **Diastolic blood pressure** |
| --- | --- | --- |
|  | **β (95% CI)** | **β (p-value)** |
| BMI | 1.96 (1.69; 2.23) | 1.28 (1.11; 1.45) |
| Sex (male vs. female) | -4.33 (-10.84; 2.19) | -1.57 (-5.75; 2.61) |
| Sex*BMI (male) | 0.88 (0.42; 1.34) | 0.31 (0.01; 0.61) |
| BMI^2^ | -0.02 (-0.03; -0.02) | -0.01 (-0.02; -0.01) |
| Sex*BMI^2^ | -0.01 (-0.02; -0.01) | -0.01 (-0.01; 0.00) |
| Age | -0.29 (-0.35; -0.23) | 0.45 (0.43; 0.48) |
| Age^2^ | 0.01 (0.01; 0.01) | -0.01 (-0.01; -0.01) |
| Education |  |  |
| 7-11 vs. <7 years | -0.28 (-0.70; 0.15) | 0.54 (0.32; 0.76) |
| 12+ vs. <7 years | -1.22 (-1.71; -0.74) | 0.75 (0.48; 1.01) |
| Socioeconomic position |  |  |
| Middle vs. low | -0.62 (-1.01; -0.23) | -0.61 (-0.84; -0.37) |
| High vs. low | -0.10 (-0.60; 0.40) | -0.24 (-0.54; 0.06) |
| Area (urban vs. rural) | 0.94 (0.56; 1.32) | 0.78 (0.53; 1.03) |
| Altitude |  |  |
| 501-2500 vs. <500 m.a.s.l. | -0.84 (-1.21; -0.47) | 0.65 (0.41; 0.88) |
| 2501+ vs. <500 m.a.s.l. | -1.84 (-2.15; -1.52) | 1.70 (1.49; 1.90) |
| Year | -0.08 (-0.17; 0.03) | 0.19 (0.14; 0.24) |
|  |  |  |
| P-value for interaction term | p < 0.001 | p < 0.001 |

**Table S3.** Association between body mass index and blood pressure by age categories:

adjusted models

| **Model variables** | **Systolic blood pressure** | **Diastolic blood pressure** |
| --- | --- | --- |
|  | **β (95% CI)** | **β (p-value)** |
| BMI | 2.14 (1.89; 2.39) | 1.13 (0.96; 1.31) |
| Age (male vs. female) |  |  |
| 40-59 vs <40 years | -12.31 (-18.84; -5.78) | -12.5 (-16.70; -8.29) |
| 60+ vs <40 years | -5.85 (-17.60; -5.90) | -0.71 (-6.89; 5.48) |
| Age*BMI (male) |  |  |
| 40-59 vs <40 years | 0.75 (0.30; 1.19) | 0.91 (0.62; 1.20) |
| 60+ vs <40 years | 0.48 (-0.35; 1.31) | 0.23 (-0.21; 0.67) |
| BMI^2^ | -0.02 (-0.03; -0.02) | -0.01 (-0.01; -0.01) |
| Age*BMI^2^ |  |  |
| 40-59 vs <40 years | -0.01 (-0.01; -0.01) | -0.02 (-0.02; -0.01) |
| 60+ vs <40 years | -0.01 (-0.02; 0.01) | -0.01(-0.01; 0.01) |
| Sex (male vs. female) | 9.91 (9.63; 10.19) | 4.84 (4.68; 5.01) |
| Age | -0.34 (-0.41; -0.27) | 0.39 (0.36; 0.43) |
| Age^2^ | 0.01 (0.01; 0.01) | -0.01 (-0.01; -0.01) |
| Education |  |  |
| 7-11 vs. <7 years | -0.28 (-0.70; 0.14) | 0.54 (0.31; 0.76) |
| 12+ vs. <7 years | -1.17 (-1.65; -0.69) | 0.79 (0.52; 1.05) |
| Socioeconomic position |  |  |
| Middle vs. low | -0.68 (-1.07; -0.29) | -0.59 (-0.82; -0.35) |
| High vs. low | -0.19 (-0.69; 0.31) | -0.18 (-0.48; 0.11) |
| Area (urban vs. rural) | 0.94 (0.55; 1.32) | 0.80 (0.54; 1.05) |
| Altitude |  |  |
| 501-2500 vs. <500 m.a.s.l. | -0.84 (-1.21; -0.47) | 0.64 (0.41; 0.88) |
| 2501+ vs. <500 m.a.s.l. | -1.85 (-2.17; -1.53) | 1.67 (1.47; 1.87) |
| Year | -0.08 (-0.17; 0.01) | 0.18 (0.13; 0.23) |
|  |  |  |
| P-value for interaction term | p < 0.001 | p < 0.001 |

**Table S4.** Association between body mass index and blood pressure by education level:

adjusted models

| **Model variables** | **Systolic blood pressure** | **Diastolic blood pressure** |
| --- | --- | --- |
|  | **β (95% CI)** | **β (p-value)** |
| BMI | 2.75 (2.33; 3.18) | 1.49 (1.26; 1.71) |
| Education |  |  |
| 7-11 vs <7 years | 6.38 (-1.49; 14.25) | -2.26 (-6.56; 2.04) |
| 12+ vs <7 years | 12.81 (4.83; 20.79) | 1.73 (-3.40; 6.87) |
| Education*BMI (male) |  |  |
| 7-11 vs <7 years | -0.34 (-0.89; 0.22) | 0.12 (-0.18; 0.43) |
| 12+ vs <7 years | -0.89 (-1.45; -0.33) | -0.17 (-0.54; 0.20) |
| BMI^2^ | -0.03 (-0.04; -0.02) | -0.02 (-0.01; 0.01) |
| Education*BMI^2^ |  |  |
| 7-11 vs <7 years | 0.01 (-0.01; 0.01) | -0.01 (-0.01; 0.01) |
| 12+ vs <7 years | 0.01 (-0.01; 0.02) | -0.01(-0.01; 0.01) |
| Sex (male vs. female) | 9.93 (9.65; 10.20) | 4.82 (4.66; 4.99) |
| Age | -0.29 (-0.35; -0.23) | 0.45 (0.42; 0.48) |
| Age^2^ | 0.01 (0.01; 0.01) | -0.01 (-0.01; -0.01) |
| Socioeconomic position |  |  |
| Middle vs. low | -0.69 (-1.08; -0.30) | -0.53 (-0.77; -0.29) |
| High vs. low | -0.13 (-0.63; 0.37) | -0.14 (-0.44; 0.15) |
| Area (urban vs. rural) | 0.92 (0.53; 1.30) | 0.82 (0.57; 1.07) |
| Altitude |  |  |
| 501-2500 vs. <500 m.a.s.l. | -0.85 (-1.22; -0.48) | 0.64 (0.40; 0.87) |
| 2501+ vs. <500 m.a.s.l. | -1.86 (-2.18; -1.54) | 1.67 (1.47; 1.87) |
| Year | -0.09 (-0.17; -0.01) | 0.19 (0.14; 0.24) |
|  |  |  |
| P-value for interaction term | p < 0.001 | p < 0.001 |

**Table S5.** Association between body mass index and blood pressure by socioeconomic position: adjusted models

| **Model variables** | **Systolic blood pressure** | **Diastolic blood pressure** |
| --- | --- | --- |
|  | **β (95% CI)** | **β (p-value)** |
| BMI | 2.45 (2.06; 2.83) | 1.05 (0.81; 1.30) |
| Socioeconomic position |  |  |
| Middle vs low | -6.40 (-13.81; 1.00) | -9.53 (-14.11; -4.95) |
| High vs. low | 5.35 (-1.81; 12.51) | -7.44 (-12.01; -2.87) |
| Education*BMI (male) |  |  |
| Middle vs low | 0.40 (-0.13; 0.92) | 0.61 (0.28; 0.94) |
| High vs. low | -0.41 (-0.91; 0.10) | 0.48 (0.15; 0.80) |
| BMI^2^ | -0.03 (-0.04; -0.02) | -0.01 (-0.01; -0.01) |
| Education*BMI^2^ |  |  |
| Middle vs low | -0.01 (-0.02; 0.01) | -0.01 (-0.02; 0.01) |
| High vs. low | 0.01 (-0.01; 0.02) | -0.01(-0.01; -0.01) |
| Sex (male vs. female) | 9.89 (9.61; 10.17) | 4.84 (4.67; 5.00) |
| Age | -0.29 (-0.35; -0.23) | 0.45 (0.42; 0.48) |
| Age^2^ | 0.01 (0.01; 0.01) | -0.01 (-0.01; -0.01) |
| Education level |  |  |
| 7-11 vs. <7 years | -0.25 (-0.67; 0.17) | 0.56 (-0.34; 0.79) |
| 12+ vs. <7 years | -1.14 (-1.62; -0.66) | 0.83 (0.56; 1.09) |
| Area (urban vs. rural) | 0.97 (0.59; 1.35) | 0.81 (0.56; 1.06) |
| Altitude |  |  |
| 501-2500 vs. <500 m.a.s.l. | -0.86 (-1.23; -0.49) | 0.63 (0.40; 0.87) |
| 2501+ vs. <500 m.a.s.l. | -1.87 (-2.19; -1.56) | 1.66 (1.46; 1.86) |
| Year | -0.08 (-0.17; 0.01) | 0.19 (0.14; 0.24) |
|  |  |  |
| P-value for interaction term | p = 0.03 | p < 0.001 |

**Table S6.** Association between body mass index and blood pressure by area:

adjusted models

| **Model variables** | **Systolic blood pressure** | **Diastolic blood pressure** |
| --- | --- | --- |
|  | **β (95% CI)** | **β (p-value)** |
| BMI | 2.46 (2.11; 2.80) | 1.10 (0.89; 1.31) |
| Area (urban vs. rural) | 1.83 (-4.17; 7.83) | -6.39 (-10.07; -2.71) |
| Area*BMI (urban) | -0.10 (-0.52; 0.33) | 0.47 (0.21; 0.74) |
| BMI^2^ | -0.03 (-0.03; -0.02) | -0.01 (-0.01; -0.01) |
| Area*BMI^2^ (urban) | 0.00 (-0.01; 0.01) | -0.01 (-0.01; -0.01) |
| Sex (male vs. female) | 9.88 (9.60; 10.16) | 4.83 (4.67; 5.00) |
| Age | -0.29 (-0.35; -0.23) | 0.45 (0.42; 0.48) |
| Age^2^ | 0.01 (0.01; 0.01) | -0.01 (-0.01; -0.01) |
| Education level |  |  |
| 7-11 vs. <7 years | -0.25 (-0.67; 0.17) | 0.56 (-0.34; 0.79) |
| 12+ vs. <7 years | -1.14 (-1.62; -0.67) | 0.83 (0.56; 1.09) |
| Socioeconomic position |  |  |
| Middle vs. low | -0.59 (-0.98; -0.20) | -0.55 (-0.79; -0.32) |
| High vs. low | -0.04 (-0.54; 0.45) | -0.18 (-0.47; 0.12) |
| Altitude |  |  |
| 501-2500 vs. <500 m.a.s.l. | -0.86 (-1.22; -0.48) | 0.63 (0.40; 0.87) |
| 2501+ vs. <500 m.a.s.l. | -1.87 (-2.19; -1.55) | 1.66 (1.46; 1.87) |
| Year | -0.08 (-0.17; 0.01) | 0.19 (0.14; 0.24) |
|  |  |  |
| P-value for interaction term | p = 0.39 | p < 0.001 |

**Table S7.** Association between body mass index and blood pressure by altitude:

adjusted models

| **Model variables** | **Systolic blood pressure** | **Diastolic blood pressure** |
| --- | --- | --- |
|  | **β (95% CI)** | **β (p-value)** |
| BMI | 2.56 (2.28; 2.84) | 1.67 (1.49; 1.84) |
| Altitude |  |  |
| 501-2500 vs. <500 masl | 11.84 (3.75; 19.93) | 10.61 (6.02; 15.21) |
| 2501+ vs. <500 masl | 5.78 (-1.46; 13.01) | 10.78 (6.15; 15.41) |
| Altitude*BMI |  |  |
| 501-2500 vs. <500 masl | -0.82 (-1.39; -0.25) | -0.67 (-1.00; -0.35) |
| 2501+ vs. <500 masl | -0.54 (-1.06; -0.02) | -0.63 (-0.97; -0.29) |
| BMI^2^ | -0.03 (-0.03; -0.02) | -0.02 (-0.02; -0.02) |
| Altitude*BMI^2^ |  |  |
| 501-2500 vs. <500 masl | 0.01 (0.01; 0.02) | 0.01 (0.01; 0.02) |
| 2501+ vs. <500 masl | 0.01 (-0.01; 0.02) | 0.01 (0.01; 0.02) |
| Sex (male vs. female) | 9.88 (9.60; 10.16) | 4.84 (4.68; 5.00) |
| Age | -0.29 (-0.35; -0.23) | 0.45 (0.42; 0.48) |
| Age^2^ | 0.01 (0.01; 0.01) | -0.01 (-0.01; -0.01) |
| Education level |  |  |
| 7-11 vs. <7 years | -0.25 (-0.67; 0.17) | 0.56 (-0.34; 0.79) |
| 12+ vs. <7 years | -1.14 (-1.62; -0.66) | 0.82 (0.56; 1.09) |
| Socioeconomic position |  |  |
| Middle vs. low | -0.58 (-0.97; -0.19) | -0.57 (-0.81; -0.33) |
| High vs. low | -0.04 (-0.53; 0.46) | -0.18 (-0.48; 0.11) |
| Area (urban vs. rural) | 0.97 (0.59; 1.35) | 0.80 (0.55; 1.05) |
| Year | -0.08 (-0.17; 0.01) | 0.19 (0.14; 0.24) |
|  |  |  |
| P-value for interaction term | p = 0.005 | p < 0.001 |

## Table S8. Estimation of blood pressure levels according to body mass index (BMI) by age group

| **BMI (Kg/m^2^)** | **<40 years** | | **40 – 59 years** | | **60+ years** | |
| --- | --- | --- | --- | --- | --- | --- |
|  | **In the overall sample** | **Only those without antihypertensive medication** | **In the overall sample** | **Only those without antihypertensive medication** | **In the overall sample** | **Only those without antihypertensive medication** |
|  | ***Systolic blood pressure (mm Hg)*** | | | | | |
| 10 | 100.1 (98.6 – 101.6) | 99.3 (97.8 – 100.8) | 94.4 (92.0 – 96.8) | 94.0 (91.7 – 96.4) | 98.7 (93.9 – 103.4) | 102.9 (97.7 – 108.0) |
| 15 | 107.6 (106.8 – 108.5) | 107.0 (106.1 – 107.8) | 104.7 (103.3 – 106.0) | 104.3 (103.0 – 105.6) | 108.1 (105.5 – 110.8) | 109.9 (107.1 – 112.7) |
| 20 | 114.0 (113.5 – 114.4) | 113.4 (112.9 – 113.8) | 113.3 (112.6 – 113.9) | 112.9 (112.3 – 113.5) | 116.2 (114.8 – 117.6) | 116.2 (114.8 – 117.6) |
| 25 | 119.0 (118.7 – 119.4) | 118.5 (118.2 – 118.8) | 120.2 (119.9 – 120.6) | 119.7 (119.4 – 120.1) | 122.8 (121.8 – 123.8) | 121.7 (120.7 – 122.7) |
| 30 | 122.9 (122.5 – 123.2) | 122.3 (122.0 – 122.7) | 125.5 (125.1 – 125.8) | 124.9 (124.5 – 125.2) | 128.0 (127.0 – 128.9) | 126.4 (125.5 – 127.4) |
| 35 | 125.4 (125.0 – 125.8) | 124.9 (124.5 – 125.3) | 129.1 (128.5 – 129.6) | 128.3 (127.8 – 128.8) | 131.7 (130.5 – 132.8) | 130.4 (129.0 – 131.8) |
| 40 | 126.8 (126.0 – 127.5) | 126.2 (125.5 – 126.9) | 131.0 (130.1 – 131.9) | 130.0 (129.1 – 131.0) | 133.9 (131.7 – 136.2) | 133.6 (130.9 – 136.4) |
| 45 | 126.8 (125.5 – 128.2) | 126.3 (125.0 – 127.6) | 131.3 (129.6 – 133.0) | 130.0 (128.3 – 131.7) | 134.8 (130.6 – 138.9) | 136.1 (131.0 – 141.2) |
| 50 | 125.7 (123.5 – 127.8) | 125.0 (122.9 – 127.1) | 129.9 (127.0 – 132.7) | 128.3 (125.4 – 131.1) | 134.2 (127.3 – 141.0) | 137.8 (129.4 – 146.1) |
|  | ***Diastolic blood pressure (mm Hg)*** | | | | | |
| 10 | 58.5 (57.5 – 59.6) | 58.4 (57.4 – 59.5) | 53.6 (52.1 – 55.1) | 53.3 (51.8 – 54.8) | 59.5 (57.1 – 61.8) | 60.8 (58.3 – 63.3) |
| 15 | 63.0 (62.4 – 63.6) | 62.9 (62.3 – 63.5) | 60.7 (59.9 – 61.6) | 60.5 (59.7 – 61.3) | 64.3 (63.0 – 65.6) | 64.8 (63.4 – 66.1) |
| 20 | 67.0 (66.7 – 67.3) | 66.9 (66.6 – 67.2) | 66.6 (66.2 – 67.0) | 66.4 (66.0 – 66.8) | 68.3 (67.6 – 68.9) | 68.2 (67.5 – 68.9) |
| 25 | 70.5 (70.3 – 70.7) | 70.4 (70.2 – 70.6) | 71.3 (71.0 – 71.5) | 71.1 (70.8 – 71.3) | 71.4 (70.9 – 71.9) | 71.1 (70.5 – 71.6) |
| 30 | 73.5 (73.3 – 73.7) | 73.3 (73.2 – 73.5) | 74.7 (74.4 – 74.9) | 74.4 (74.2 – 74.7) | 73.8 (73.3 – 74.3) | 73.4 (72.9 – 73.9) |
| 35 | 76.0 (75.8 – 76.3) | 75.8 (75.6 – 76.1) | 76.9 (76.5 – 77.2) | 76.5 (76.2 – 76.8) | 75.4 (74.8 – 76.0) | 75.2 (74.5 – 75.9) |
| 40 | 78.1 (77.6 – 78.6) | 77.9 (77.3 – 78.4) | 77.8 (77.2 – 78.4) | 77.3 (76.7 – 77.9) | 76.1 (75.0 – 77.3) | 76.5 (75.0 – 77.9) |
| 45 | 79.6 (78.7 – 80.6) | 79.4 (78.4 – 80.3) | 77.5 (76.4 – 78.6) | 76.8 (75.7 – 77.9) | 76.1 (73.9 – 78.2) | 77.2 (74.5 – 79.8) |
| 50 | 80.7 (79.2 – 82.3) | 80.4 (78.9 – 81.9) | 76.0 (74.2 – 77.7) | 75.0 (73.2 – 76.9) | 75.2 (71.6 – 78.8) | 77.4 (73.1 – 81.6) |

## Table S9. Estimation of blood pressure levels according to body mass index (BMI) by education level (in years)

| **BMI (Kg/m^2^)** | **<7 years** | | **7 – 11 years** | | **12+ years** | |
| --- | --- | --- | --- | --- | --- | --- |
|  | **In the overall sample** | **Only those without antihypertensive medication** | **In the total sample** | **Only those without antihypertensive medication** | **In the total sample** | **Only those without antihypertensive medication** |
|  | ***Systolic blood pressure (mm Hg)*** | | | | | |
| 10 | 95.7 (93.1 – 98.2) | 97.2 (94.7 – 99.7) | 99.0 (96.8 – 101.2) | 98.5 (96.4 – 100.6) | 100.9 (98.7 – 103.2) | 100.1 (97.9 – 102.3) |
| 15 | 105.5 (104.1 – 106.9) | 106.0 (104.7 – 107.4) | 107.5 (106.4 – 108.7) | 107.0 (105.9 – 108.2) | 108.0 (106.8 – 109.2) | 107.3 (106.1 – 108.5) |
| 20 | 113.7 (113.1 – 114.3) | 113.5 (112.9 – 114.1) | 114.7 (114.1 – 115.2) | 114.1 (113.6 – 114.6) | 114.1 (113.6 – 114.6) | 113.5 (113.0 – 114.0) |
| 25 | 120.3 (119.9 – 120.7) | 119.6 (119.3 – 119.9) | 120.4 (120.1 – 120.6) | 119.8 (119.5 – 120.0) | 119.3 (119.0 – 119.6) | 118.8 (118.5 – 119.0) |
| 30 | 125.4 (124.9 – 125.8) | 124.3 (123.9 – 124.7) | 124.7 (124.3 – 125.0) | 124.0 (123.7 – 124.3) | 123.6 (123.3 – 124.0) | 123.0 (122.7 – 123.4) |
| 35 | 128.8 (128.2 – 129.4) | 127.7 (127.1 – 128.3) | 127.5 (127.0 – 128.0) | 126.7 (126.3 – 127.2) | 127.0 (126.5 – 127.6) | 126.3 (125.8 – 126.9) |
| 40 | 130.7 (129.5 – 131.8) | 129.7 (128.6 – 130.8) | 129.0 (128.0 – 129.9) | 128.1 (127.2 – 128.9) | 129.5 (128.4 – 130.6) | 128.7 (127.6 – 129.7) |
| 45 | 131.0 (128.8 – 133.1) | 130.3 (128.3 – 132.4) | 129.0 (127.2 – 130.8) | 127.9 (126.2 – 129.6) | 131.1 (129.1 – 133.0) | 130.1 (128.1 – 132.0) |
| 50 | 129.7 (126.2 – 133.1) | 129.6 (126.2 – 133.1) | 127.6 (124.7 – 130.6) | 126.3 (123.5 – 129.1) | 131.7 (128.6 – 134.9) | 130.4 (127.3 – 133.6) |
|  | ***Diastolic blood pressure (mm Hg)*** | | | | | |
| 10 | 57.4 (56.0 – 58.7) | 57.9 (56.5 – 59.2) | 56.3 (55.0 – 57.5) | 56.3 (55.1 – 57.6) | 57.9 (56.2 – 59.6) | 57.5 (55.7 – 59.2) |
| 15 | 62.6 (61.9 – 63.4) | 62.8 (62.1 – 63.5) | 62.1 (61.4 – 62.8) | 62.0 (61.3 – 62.7) | 62.9 (62.0 – 63.8) | 62.6 (61.7 – 63.5) |
| 20 | 67.0 (66.7 – 67.4) | 67.0 (66.6 – 67.3) | 66.9 (66.6 – 67.3) | 66.8 (66.5 – 67.1) | 67.3 (66.9 – 67.7) | 67.1 (66.7 – 67.4) |
| 25 | 70.6 (70.4 – 70.8) | 70.4 (70.2 – 70.6) | 70.9 (70.8 – 71.1) | 70.8 (70.6 – 70.9) | 71.1 (70.9 – 71.3) | 70.9 (70.7 – 71.0) |
| 30 | 73.2 (73.0 – 73.5) | 73.0 (72.8 – 73.2) | 74.0 (73.8 – 74.2) | 73.8 (73.6 – 74.0) | 74.2 (74.0 – 74.4) | 74.0 (73.8 – 74.2) |
| 35 | 75.0 (74.7 – 75.3) | 74.9 (74.6 – 75.2) | 76.2 (75.9 – 76.5) | 76.0 (75.7 – 76.3) | 76.7 (76.4 – 77.1) | 76.5 (76.1 – 76.8) |
| 40 | 75.9 (75.3 – 76.6) | 76.0 (75.3 – 76.7) | 77.5 (76.9 – 78.0) | 77.3 (76.7 – 77.8) | 78.6 (77.8 – 79.4) | 78.3 (77.5 – 79.1) |
| 45 | 76.0 (74.8 – 77.1) | 76.4 (75.1 – 77.6) | 77.9 (76.8 – 78.9) | 77.7 (76.6 – 78.7) | 79.9 (78.4 – 81.4) | 79.5 (77.9 – 81.0) |
| 50 | 75.2 (73.3 – 77.1) | 75.9 (73.9 – 77.9) | 77.3 (75.6 – 79.0) | 77.2 (75.5 – 78.9) | 80.5 (78.0 – 83.0) | 79.9 (77.3 – 82.5) |

## Table S10. Estimation of blood pressure levels according to body mass index (BMI) by socioeconomic position

| **BMI (Kg/m^2^)** | **Low** | | **Middle** | | **High** | |
| --- | --- | --- | --- | --- | --- | --- |
|  | **In the overall sample** | **Only those without antihypertensive medication** | **In the total sample** | **Only those without antihypertensive medication** | **In the total sample** | **Only those without antihypertensive medication** |
|  | ***Systolic blood pressure (mm Hg)*** | | | | | |
| 10 | 98.4 (96.2 – 100.7) | 99.8 (97.5 – 102.0) | 95.4 (93.1 – 97.6) | 95.0 (92.8 – 97.2) | 100.5 (98.4 – 102.6) | 100.2 (98.1 – 102.3) |
| 15 | 107.1 (106.0 – 108.3) | 107.7 (106.5 – 108.9) | 105.2 (104.0 – 106.5) | 104.8 (103.6 – 105.9) | 108.1 (106.9 – 109.3) | 107.7 (106.5 – 108.8) |
| 20 | 114.4 (113.9 – 114.9) | 114.4 (113.8 – 114.9) | 113.3 (112.8 – 113.9) | 112.8 (112.3 – 113.3) | 114.6 (114.1 – 115.2) | 114.1 (113.5 – 114.6) |
| 25 | 120.3 (119.9 – 120.6) | 119.8 (119.5 – 120.2) | 119.7 (119.4 – 120.0) | 119.1 (118.8 – 119.3) | 120.1 (119.8 – 120.4) | 119.4 (119.2 – 119.7) |
| 30 | 124.7 (124.3 – 125.1) | 124.0 (123.6 – 124.4) | 124.3 (124.0 – 124.6) | 123.6 (123.3 – 123.9) | 124.6 (124.2 – 124.9) | 123.8 (123.5 – 124.1) |
| 35 | 127.7 (127.1 – 128.4) | 127.0 (126.4 – 127.6) | 127.2 (126.7 – 127.6) | 126.4 (125.9 – 126.8) | 127.9 (127.5 – 128.4) | 127.0 (126.6 – 127.5) |
| 40 | 129.3 (128.1 – 130.5) | 128.8 (127.6 – 129.9) | 128.3 (127.3 – 129.2) | 127.4 (126.4 – 128.3) | 130.3 (129.4 – 131.1) | 129.3 (128.4 – 130.1) |
| 45 | 129.5 (127.3 – 131.6) | 129.3 (127.1 – 131.4) | 127.6 (125.8 – 129.5) | 126.7 (124.9 – 128.5) | 131.6 (130.0 – 133.1) | 130.4 (128.9 – 132.0) |
| 50 | 128.2 (124.8 – 131.7) | 128.6 (125.1 – 132.0) | 125.2 (122.2 – 128.3) | 124.2 (121.2 – 127.2) | 131.8 (129.3 – 134.4) | 130.6 (128.0 – 133.1) |
|  | ***Diastolic blood pressure (mm Hg)*** | | | | | |
| 10 | 60.1 (58.8 – 61.5) | 60.5 (59.1 – 61.9) | 55.7 (54.3 – 57.0) | 55.7 (54.3 – 57.0) | 56.7 (55.4 – 58.1) | 56.5 (55.1 – 57.9) |
| 15 | 64.2 (63.5 – 65.0) | 64.4 (63.6 – 65.1) | 61.6 (60.9 – 62.3) | 61.5 (60.8 – 62.2) | 62.3 (61.5 – 63.1) | 62.1 (61.3 – 62.8) |
| 20 | 67.9 (67.6 – 68.2) | 67.9 (67.5 – 68.2) | 66.6 (66.3 – 66.9) | 66.4 (66.1 – 66.7) | 67.0 (66.7 – 67.4) | 66.8 (66.5 – 67.2) |
| 25 | 71.1 (70.9 – 71.3) | 71.0 (70.7 – 71.2) | 70.6 (70.4 – 70.7) | 70.4 (70.2 – 70.5) | 70.9 (70.8 – 71.1) | 70.7 (70.6 – 70.9) |
| 30 | 73.9 (73.6 – 74.1) | 73.7 (73.4 – 73.9) | 73.7 (73.5 – 73.8) | 73.4 (73.2 – 73.6) | 74.0 (73.8 – 74.2) | 73.8 (73.6 – 74.0) |
| 35 | 76.2 (75.8 – 76.6) | 76.0 (75.6 – 76.4) | 75.8 (75.5 – 76.0) | 75.6 (75.3 – 75.8) | 76.2 (76.0 – 76.5) | 76.1 (75.8 – 76.3) |
| 40 | 78.0 (77.2 – 78.8) | 77.9 (77.1 – 78.7) | 76.9 (76.4 – 77.5) | 76.8 (76.2 – 77.4) | 77.6 (77.1 – 78.2) | 77.4 (76.9 – 78.0) |
| 45 | 79.4 (78.0 – 80.8) | 79.4 (77.9 – 81.0) | 77.1 (76.1 – 78.2) | 77.1 (76.0 – 78.2) | 78.2 (77.2 – 79.2) | 78.0 (76.9 – 79.1) |
| 50 | 80.3 (78.0 – 82.6) | 80.6 (78.1 – 83.1) | 76.4 (74.6 – 78.2) | 76.5 (74.6 – 78.3) | 77.9 (76.3 – 79.6) | 77.7 (76.0 – 79.5) |

## Table S11. Estimation of blood pressure levels according to body mass index (BMI) by study area

| **BMI (Kg/m^2^)** | **Rural** | | **Urban** | |
| --- | --- | --- | --- | --- |
|  | **In the overall sample** | **Only those without antihypertensive medication** | **In the total sample** | **Only those without antihypertensive medication** |
|  | ***Systolic blood pressure (mm Hg)*** | | | |
| 10 | 97.6 (95.6 – 99.6) | 98.3 (96.3 – 100.4) | 98.7 (97.1 – 100.3) | 98.4 (96.9 – 100.0) |
| 15 | 106.3 (105.2 – 107.4) | 106.4 (105.3 – 107.5) | 107.2 (106.3 – 108.1) | 106.8 (106.0 – 107.7) |
| 20 | 113.5 (113.0 – 114.0) | 113.2 (112.7 – 113.7) | 114.4 (114.0 – 114.8) | 113.9 (113.5 – 114.2) |
| 25 | 119.3 (119.0 – 119.7) | 118.7 (118.4 – 119.0) | 120.2 (120.0 – 120.4) | 119.6 (119.4 – 119.8) |
| 30 | 123.7 (123.3 – 124.1) | 122.9 (122.5 – 123.3) | 124.7 (124.5 – 125.0) | 124.0 (123.8 – 124.3) |
| 35 | 126.7 (126.1 – 127.2) | 125.8 (125.2 – 126.3) | 128.0 (127.6 – 128.3) | 127.1 (126.8 – 127.5) |
| 40 | 128.2 (127.2 – 129.1) | 127.3 (126.3 – 128.3) | 129.9 (129.2 – 130.5) | 128.9 (128.3 – 129.6) |
| 45 | 128.2 (126.5 – 130.0) | 127.6 (125.8 – 129.4) | 130.4 (129.2 – 131.6) | 129.4 (128.1 – 130.6) |
| 50 | 126.9 (124.0 – 129.7) | 126.6 (123.6 – 129.5) | 129.7 (127.6 – 131.7) | 128.5 (126.5 – 130.6) |
|  | ***Diastolic blood pressure (mm Hg)*** | | | |
| 10 | 59.0 (57.8 – 60.2) | 59.1 (57.8 – 68.4) | 56.6 (55.6 – 57.6) | 56.5 (55.5 – 57.5) |
| 15 | 63.3 (62.6 – 63.9) | 63.2 (62.6 – 63.9) | 62.3 (61.8 – 62.8) | 62.2 (61.6 – 62.7) |
| 20 | 67.0 (66.7 – 67.3) | 66.9 (66.6 – 67.2) | 67.1 (66.9 – 67.4) | 66.9 (66.7 – 67.2) |
| 25 | 70.3 (70.0 – 70.5) | 70.0 (69.8 – 70.3) | 71.1 (70.9 – 71.2) | 70.9 (70.7 – 71.0) |
| 30 | 73.0 (72.8 – 73.2) | 72.7 (72.5 – 73.0) | 74.1 (74.0 – 74.3) | 73.9 (73.8 – 74.1) |
| 35 | 75.3 (74.9 – 75.6) | 75.0 (74.6 – 75.3) | 76.3 (76.1 – 76.5) | 76.1 (75.9 – 76.4) |
| 40 | 77.0 (76.4 – 77.6) | 76.7 (76.0 – 77.4) | 77.6 (77.2 – 78.0) | 77.5 (77.1 – 77.9) |
| 45 | 78.2 (77.1 – 79.4) | 78.0 (76.8 – 79.3) | 78.1 (77.3 – 78.8) | 78.0 (77.2 – 78.8) |
| 50 | 79.0 (77.1 – 80.8) | 78.8 (76.8 – 80.8) | 77.6 (76.4 – 78.8) | 77.6 (76.3 – 78.9) |

## Table S12. Estimation of blood pressure levels according to body mass index (BMI) by altitude

| **BMI (Kg/m^2^)** | **≤500 m.a.s.l.** | | **501 – 2500 m.a.s.l.** | | **≥2501 m.a.s.l** | |
| --- | --- | --- | --- | --- | --- | --- |
|  | **In the overall sample** | **Only those without antihypertensive medication** | **In the overall sample** | **Only those without antihypertensive medication** | **In the overall sample** | **Only those without antihypertensive medication** |
|  | ***Systolic blood pressure (mm Hg)*** | | | | | |
| 10 | 97.7 (95.9 – 99.4) | 97.5 (95.8 – 99.2) | 102.6 (99.6 – 105.6) | 101.8 (99.0 – 104.7) | 99.0 (96.5 – 101.4) | 100.5 (97.9 – 103.0) |
| 15 | 106.8 (105.8 – 107.8) | 106.4 (105.5 – 107.4) | 109.2 (107.5 – 110.8) | 108.6 (107.0 – 110.1) | 106.5 (105.3 – 107.8) | 107.2 (105.9 – 108.4) |
| 20 | 114.4 (114.0 – 114.8) | 113.9 (113.4 – 114.3) | 114.9 (114.2 – 115.6) | 114.3 (113.7 – 115.0) | 113.1 (112.6 – 113.6) | 113.1 (112.6 – 113.5) |
| 25 | 120.5 (120.3 – 120.8) | 119.8 (119.6 – 120.0) | 119.8 (119.4 – 120.1) | 119.2 (118.9 – 119.6) | 118.6 (118.4 – 118.9) | 118.2 (118.0 – 118.5) |
| 30 | 125.2 (124.9 – 125.4) | 124.3 (124.1 – 124.6) | 123.8 (123.4 – 124.2) | 123.2 (122.8 – 123.6) | 123.1 (122.8 – 123.5) | 122.7 (122.4 – 123.0) |
| 35 | 128.3 (128.0 – 128.7) | 127.3 (127.0 – 127.7) | 126.9 (126.3 – 127.6) | 126.2 (125.6 – 126.8) | 126.6 (126.0 – 127.3) | 126.4 (125.7 – 127.0) |
| 40 | 130.0 (129.3 – 130.8) | 128.9 (128.2 – 129.6) | 129.3 (128.0 – 130.5) | 128.4 (127.2 – 129.5) | 129.1 (127.8 – 130.5) | 129.3 (127.8 – 130.8) |
| 45 | 130.2 (128.9 – 131.6) | 129.0 (127.6 – 130.3) | 130.7 (128.3 – 133.1) | 129.6 (127.4 – 131.7) | 130.6 (128.0 – 133.2) | 131.5 (128.7 – 134.2) |
| 50 | 129.0 (126.7 – 131.2) | 127.6 (125.4 – 129.7) | 131.3 (127.3 – 135.3) | 129.9 (126.3 – 133.5) | 131.1 (126.9 – 135.3) | 132.9 (128.4 – 137.4) |
|  | ***Diastolic blood pressure (mm Hg)*** | | | | | |
| 10 | 55.4 (54.3 – 56.5) | 55.2 (54.1 – 56.3) | 60.4 (58.7 – 62.0) | 60.2 (58.5 – 61.8) | 60.9 (59.3 – 62.5) | 61.6 (60.0 – 63.1) |
| 15 | 61.3 (60.7 – 61.9) | 61.1 (60.5 – 61.7) | 64.3 (63.4 – 65.2) | 64.2 (63.3 – 65.1) | 65.0 (64.2 – 65.8) | 65.3 (64.5 – 66.1) |
| 20 | 66.3 (66.1 – 66.6) | 66.1 (65.9 – 66.4) | 67.9 (67.5 – 68.3) | 67.7 (67.3 – 68.1) | 68.7 (68.4 – 69.0) | 68.7 (68.4 – 69.0) |
| 25 | 70.4 (70.3 – 70.5) | 70.2 (70.1 – 70.3) | 71.0 (70.8 – 71.3) | 70.9 (70.7 – 71.1) | 72.0 (71.9 – 72.2) | 71.9 (71.7 – 72.0) |
| 30 | 73.5 (73.4 – 73.7) | 73.3 (73.1 – 73.5) | 73.8 (73.6 – 74.0) | 73.6 (73.4 – 73.9) | 74.9 (74.7 – 75.1) | 74.7 (74.5 – 74.9) |
| 35 | 75.7 (75.4 – 75.9) | 75.5 (75.2 – 75.7) | 76.1 (75.8 – 76.5) | 75.9 (75.6 – 76.3) | 77.3 (76.9 – 77.7) | 77.3 (76.9 – 77.7) |
| 40 | 76.9 (76.4 – 77.3) | 76.7 (76.2 – 77.1) | 78.1 (77.4 – 78.8) | 77.8 (77.1 – 78.6) | 79.4 (78.4 – 80.3) | 79.5 (78.7 – 80.4) |
| 45 | 77.1 (76.3 – 78.0) | 76.9 (76.1 – 77.8) | 79.6 (78.3 – 81.0) | 79.3 (77.9 – 80.8) | 81.0 (79.2 – 82.7) | 81.5 (79.8 – 83.2) |
| 50 | 76.4 (75.1 – 77.8) | 76.2 (74.8 – 77.7) | 80.8 (78.5 – 83.0) | 80.4 (78.1 – 82.7) | 82.1 (79.3 – 85.0) | 83.2 (80.5 – 86.0) |

m.a.s.l. = meters above the sea level
